# An Unusual Presentation of Galactosemia: Hemophagocytic Lymphohistiocytosis

**DOI:** 10.5505/tjh.2012.65148

**Published:** 2012-12-05

**Authors:** Ahmet Afşin Kundak, Ayşegül Zenciroğlu, Neşe Yaralı, Belma Saygılı Karagöl, Arzu Dursun, Selim Gökçe, Nilgün Karadağ, Nurullah Okumuş

**Affiliations:** 1 Dr.Sami Ulus Hospital, Neonatology Department, Ankara, Turkey; 2 Ankara Pediatric Hematology Oncology Training and Research Hospital, Ankara, Turkey; 3 Dr.Sami Ulus Hospital, Gastroenterology Department, Ankara, Turkey

**Keywords:** Hemophagocytic lymphohistiocytosis, Galactosemia, newborn

## Abstract

Hemophagocytic lymphohistiocytosis (HLH) is a rare life-threatening condition. Uncontrolled proliferation of activated lymphocytes secreting high amounts of inflammatory cytokines seems to be the main pathogenesis. The diagnosis of HLH can often be difficult. It may presents in many forms such as fever of unknown origin, hepatitis, acute liver failure, and sepsis-like illness. Here we present a newborn galactosemia case presented with HLH. Close monitoring of the diagnostic criteria of HLH during the course of galactosemia-associated hemophagocytosis, both before and after dietary treatment, should be performed in order to fully determine if the triggering mechanism is infection or accumulation of metabolites.

**Conflict of interest:**None declared.

## Abstract (tr)

Hemofagositik lenfohistiyositozis (HLH), nadir görülen, yaşamı tehdit eden bir durumdur. Patogenezinde; yüksek miktarda sitokin üreten aktive lenfositlerin kontrolsüz proliferasyonunun olduğu düşünülmektedir. HLH tanısını koymak genellikle güçtür. Hastalar nedeni bilinmeyen ateş, hepatit, akut karaciğer yetmezliği, sepsis gibi çok farklı klinik bulgularla başvurabilir. Burada HLH ile başvuran galaktozemili bir yenidoğan vakasını sunduk. Galaktozemi ile ilişkili HLH vakalarında tanı kriterlerinin diyet tedavisi öncesi ve sonrasında yakın takip edilmesi hastalığı tetikleyen mekanizmanın enfeksiyon mu yoksa metabolit birikimi mi olduğunu daha iyi açıklayabilmek için gereklidir.

## INTRODUCTION

Hemophagocytic lymphohistiocytosis (HLH) is a rare life-threatening condition characterized by severe multisystem hyperinflammation [1]. The term HLH does not refer to a specific disease, as it has many genetic and acquired causes. Primary HLH, also known as familial HLH (FHL), is an inherited autosomal recessive disorder that occurs more commonly in cases of parental consanguinity. Secondary or acquired HLH occurs following strong immunologic activation, such as immunodeficiency, systemic infection, or malignancy. Both forms are characterized by abnormal activation of T-lymphocytes and macrophages. Uncontrolled proliferation of activated lymphocytes that secrete large quantities of inflammatory cytokines is thought to be the primary pathogenesis [2]. 

FHL is diagnosed either via mutation analysis of genes, including perforin, UNC 13D, syntaxin 11, and syntaxinbinding protein 2, or via family history. HLH can be diagnosed based on fulfillment of 5 of the following 8 clinical/ biochemical changes: fever, splenomegaly, cytopenias, hypertriglyceridemia/hypofibrinogenemia, hemophagocytosis in bone marrow, abnormal natural killer cell function assay, elevated soluble IL-2R alpha level, and elevated ferritin level. These 8 HLH criteria can also be observed in both FHL and secondary HLH [3]. Acquired HLH is primarily thought to be associated with several infectious agents, including viral, bacterial, fungal, and parasitic pathogens [4,5,6,7], and is also associated with autoimmune diseases, malignancies, and metabolic diseases [8]. Herein we present a full-term newborn with galactosemia that presented with HLH. HLH associated with galactosemia is rarely reported.

## CASE REPORT

A 3-d-old full-term girl born to consanguineous (firstdegree cousins) parents was admitted to our neonatal intensive care unit with jaundice and fever (37.6 °C). Her birth weight was normal (3300 g) and had jaundice and poor feeding 1 day prior to admission. She was the offspring of the mother’s second gravida. The first gravida was terminated via abortion. Prenatal history, mother’s laboratory findings, and family history were unremarkable. The patient was delivered via Caesarian section and had normal APGAR scores.

On physical examination she weighed 3000 g and her vital signs were stable. She had icterus, lethargy, and weak sucking. The remainder of the physical examination was unremarkable. Initial laboratory findings were as follows: white blood cell (WBC) count: 10.3 x 10^9^/L; hemoglobin (Hb): 15.7 g/dL; red blood cell (RBC) count: 4.68 x 10^12^/L; platelet (Plt) count: 233x10^9^/L; C-reactive protein (CRP): 3.34 mg/dL; total bilirubin: 15.6 mg/dL: direct bilirubin: 1 mg/dL; aspartate aminotransferase (AST): 1367 U/L; alanine aminotransferase (ALT): 504 U/L; prothrombin time (PT): >60 s; international normalized ratio (INR): >6; activated partial thromboplastin time (aPTT): 88 s. The blood of the patient and her mother was O Rh +; Coombs test was negative. Serum sodium, potassium, and creatinine were 147 mEq/L, 4.5 mEq/L, and 0.46 mg/dL, respectively. Microbiological work-up, including blood culture, was performed and ampicillin-gentamicin was empirically started. 

The patient received supportive treatment with vitamin K and fresh frozen plasma due to acute liver failure (ALF). The patient’s clinical condition deteriorated on d 2 of hospitalization and her body temperature increased to 39 °C. Due to upper gastrointestinal bleeding oral feeding was stopped. The following screening tests performed due to elevated liver enzymes and coagulopathy were negative: Epstein-Barr virus, toxoplasma, rubella, cytomegalovirus, herpes simplex virus, human immunodeficiency virus, human parvovirus B19, treponema pallidum, and hepatitis A, B, and C. The patient’s ferritin level was 5644 ng/ mL, and her fibrinogen level was 147 mg/dL, despite fresh frozen plasma treatment. During this period the patient was given an anti-oxidant cocktail that included selenium, n- acetylcysteine, vitamin E, and desferrioxamine due to the possibility of hemochromatosis, but there was no improvement in clinical or laboratory parameters. For the differential diagnosis of neonatal hemochromatosis buccal biopsy was performed, but no evidence of hemochromatosis was observed. 

**Figure 1:**
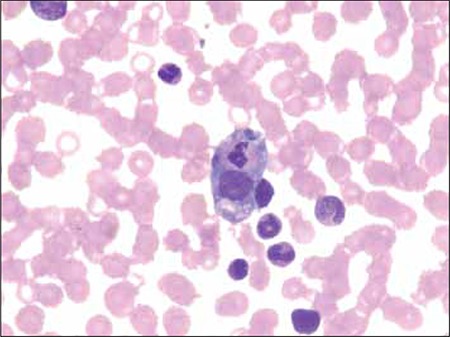
Hemophagocytosis in bone marrow aspirate.

On d 12 of hospitalization hepatosplenomegaly was noted on physical examination and the patient’s WBC count, Hb, and Plt count decreased to 3.8 x 10^9^/L, 6.5 g/ dL, and 48 x 10^9^/L, respectively. Hemophagocytosis was observed in her bone marrow aspirate (Figure 1) and CRP increased to 19 mg/dL. The patient was diagnosed as HLH based on fulfillment of 6 diagnostic criteria (fever, hemophagocytosis, hypofibrinogenemia, pancytopenia, splenomegaly, and hyperferritinemia). Antibiotic treatment was changed to imipenem due to suspected sepsis, and then vancomycin was added. Blood cultures were negative and the patient was given high-dose (1 mg/kg) intravenous immunoglobulin (IVIG) to stop the inflammatory cascade, because diverse bacterial infections were suspected to have caused HLH. Her work-up for inborn errors of metabolism showed non-specific changes, except for urine, which was positive for reducing substance; however, thin layer sugar chromatography was negative on 3 separate occasions. Liver histology showed focal hepatic necrosis, macrovesicular steatosis, vacuolar degeneration, and canalicular cholestasis. 

Due to the possibility of galactosemia, lactose-free formula was commenced together with the second dose of IVIG, after which time the patient’s clinical and laboratory findings improved. As galactose-1-phosphate uridyl transferase (GALT) enzyme analysis would have been noninformative due to previous erythrocyte transfusion during hospitalization, her GALT enzyme level was evaluated at age 3 months (72 U/L [n > 262]) and the diagnosis of galactosemia was confirmed. At age 5 months the patient’s physical examination and laboratory findings were normal.

## DISCUSSION

We described a neonatal case of galactosemia with secondary HLH and ALF. The patient recovered following IVIG treatment and a galactose-free diet. HLH is a rare and fatal disorder resulting from uncontrolled proliferation of activated lymphocytes and histiocytes that secrete large quantities of inflammatory cytokines. HLH is a clinical syndrome, rather than a single disease, associated with a variety of underlying conditions [9]. The underlying mechanism of secondary HLH is not fully understood and it can manifest at any age. It is observed in the context of infection, underlying autoimmune disorders, and some metabolic disorders [7,9]. To the best of our knowledge the presented case is only the third report of HLH secondary to galactosemia [10,11]. Previously described cases also had sepsis (confirmed via blood culture). 

The mechanism of HLH is not clear in metabolic disorders; it could be associated with tissue damage, and impaired lymphocyte and histiocyte functions, or activation of macrophages via accumulation of metabolites in some way [8,12]. In the presented case the underlying endogenous mechanism may have been tissue damage caused by galactose or its metabolites; exogenous agents such as gram-negative microorganisms that could have had an additive effect were not detected in blood culture. In galactosemic neonates the results of tests for evaluating neutrophil function, including chemiluminescence, chemotaxis, and adherence, were significantly low due to galactosemiainduced immune deficiency [13,14]. 

The diagnosis of HLH can often be difficult, as it may present in many forms, including fever of unknown origin, hepatitis, acute liver failure, and sepsis-like illness [15]. HLH diagnostic criteria may not be observed initially, as in the present case and, as such, it is important to carefully follow the clinical signs and laboratory markers of HLH during the course of illness. Acute systemic immune activation and specific findings, such as cytopenias, decreased fibrinogen or increased triglycerides, hemophagocytosis, and elevated ferritin, are criteria that differentiate HLH from other inflammatory disorders [3]. The presented case was difficult to diagnose; hemochromatosis was an initial consideration due to ALF with hyperferritinemia, and both buccal biopsy and liver biopsy were performed to confirm the diagnosis. Moreover, the presented case gradually developed the diagnostic criteria of HLH (cytopenia and splenomegaly) during the course of the disease, which were not initially observed. A lactose-free diet and high-dose IVIG stopped the hyperinflammation cascade in the presented case; the elevated liver enzymes may have been due to galactosemia or HLH. 

The principle challenge in treating patients with HLH is to diagnose as early as possible. It is also critical to search for and treat underlying triggers of HLH, and to begin specific therapy [9]. Marcoux et al. reported a neonate with a favorable outcome following antibiotic and corticosteroid treatment [10]. Treatment with IVIG, as in the presented case, or corticosteroids, as in the case reported by Marcoux et al. [10] might have an effect on satisfactory outcome and stop the hyperinflammatory process. Additionally, it is important to block triggering of the hyperinflammatory process via a lactose-free diet. As the presented case was clinically unstable, the response specific to the lactose-free diet could not be determined. 

In secondary HLH cases conditions leading to the hyperinflammatory process are generally exogenous agents, such as infections, endogenous products causing tissue damage, rheumatic diseases, and malignant diseases [9]. Sepsis-associated secondary HLH should be considered in the differential diagnosis of HLH, especially in neonates [16]. In cases of galactosemia, overlap of tissue damage and the potential risk of gram-negative infection might predispose patients to HLH. In cases of severe galactosemia with sepsis that do not respond to antibiotic treatment in addition to standard therapy the presence of HLH should be investigated. Diagnosis of FHL was not possible in the presented case. HLH improves following treatment of galactosemia and administration of IVIG, which indicates the presence of secondary HLH. 

In conclusion, it is difficult to differentiate neonatal HLH from metabolic disease and sepsis; however, differentiation of these entities should be performed. In neonates with an acute presentation, with sepsis and ALF, metabolic diseases should also be investigated, especially in regions in which consanguineous marriage is common, as in Turkey. In the literature three reports of HLH cases with galactosemia are remarkable. Close monitoring of the diagnostic criteria of HLH during the course of galactosemia- associated hemophagocytosis, both before and after dietary treatment, should be performed in order to fully determine if the triggering mechanism is infection or accumulation of metabolites.
